# Evaluating the Accuracy of Non-invasive Pulsed-Wave Doppler Echocardiography in Measuring Fetal QT and PR Intervals: A Cross-sectional Study

**DOI:** 10.2174/0115734056466578260728092631

**Published:** 2026-02-25

**Authors:** Feisal Rahimpour, Lida Jarahi, Hassan Birjandi, Mitra Ahmadian, Rasoul Raesi

**Affiliations:** 1 Department of Pediatric Cardiologist, Interventional Electrophysiologist, Mashhad Cardiovascular Medical and Research Center, Mashhad University of Medical Sciences, Mashhad, Iran; 2 Department, Community Medicine, Mashhad University of Medical Sciences, Mashhad, Iran; 3 Department of Pediatric Cardiologist, Mashhad University of Medical Sciences, Mashhad, Iran; 4 Department of Pediatric, Pediatrics Resident, Mashhad University of Medical Sciences, Mashhad, Iran; 5 Department of Public Health, School of Health, Torbat Jam Faculty of Medical Sciences, Torbat Jam, Iran

**Keywords:** QT interval, PR interval, Fetal echocardiography, Magnetocardiography, Congenital long QT syndrome, Fetal arrhythmia

## Abstract

**Introduction:**

Congenital Long QT Syndrome (LQTS) is a common cause of potentially life-threatening ventricular arrhythmias and sudden cardiac death in infants and children. Fetal Magnetocardiography (fMCG) is available in only a few specialized centers worldwide and offers accurate measurements of the QT and PR intervals. The aim of this study was to assess the diagnostic accuracy of PW Doppler echocardiographic measurement of fetal QT and PR intervals compared with the available normative data for fMCG.

**Methods:**

576 pregnant women with singleton pregnancies (16-38 weeks' gestation) were studied in this cross-sectional study between 2022 and 2023 with detailed fetal echocardiograms. Standardized pulsed-wave Doppler techniques were used to measure the PR and QT intervals. Results were compared with published normative data for fMCG, adjusted for gestational age, and were also compared with contemporaneous fMCG in a subset of 42 fetuses.

**Results:**

Mean QT interval (echo) was 270.0 ± 38.8 and mean PR interval was 119.3 ± 16.2. In the paired subset (n = 42), strong agreement was observed (PR ICC = 0.89 [95% CI: 0.81-0.94]; QT ICC = 0.82 [95% CI: 0.72-0.89]), with mean biases of +2.1 ms and +6.8 ms on Bland-Altman analysis, respectively. Sensitivity and specificity for the full cohort for prolonged intervals (> 95th percentile of GA-adjusted fMCG norms) were 85% and 78% (PPV 68%, NPV 90%). There were no clinically significant differences for PR interval or fetal heart rate, and there was no significant correlation between measured intervals and gestational age (*p* > 0.05).

**Discussion:**

Pulsed-wave Doppler echocardiography is a reliable method for measuring fetal QT and PR intervals and is in good agreement with fMCG.

**Conclusion:**

It is a practical, non-invasive, and easily accessible substitute for fMCG when it is not available for prenatal screening of cardiac repolarization and conduction abnormalities.

## 1 INTRODUCTION

Congenital Long QT Syndrome (LQTS) is an inherited cardiac channelopathy characterized by delayed ventricular repolarization, resulting in prolongation of the corrected QT (QTc) interval and an increased risk of life-threatening ventricular arrhythmias and sudden cardiac death [1-3]. The estimated prevalence of congenital LQTS is approximately 1 in 2,000 live births, corresponding to nearly 0.05% of the general population. Diagnosis is primarily based on the detection of QTc prolongation using standard electrocardiography (ECG), while Fetal Magnetocardiography (fMCG) provides a specialized noninvasive approach for evaluating fetal cardiac electrophysiology. Despite advances in diagnostic techniques, prenatal identification of LQTS remains challenging, and delayed diagnosis may result in severe clinical consequences across all age groups [1,4,5]. Conventional prenatal imaging modalities, including standard ultrasonography and pulsed-wave Doppler echocardiography, have limited capability for directly assessing fetal cardiac electrical activity. Reliable fetal ECG acquisition is hindered by the low amplitude of fetal cardiac signals and signal attenuation caused by the maternal abdominal wall, amniotic fluid, vernix caseosa, and other surrounding tissues. Consequently, accurate and reproducible assessment of fetal cardiac conduction using conventional electrical recordings remains difficult [5-8].

Over the past two decades, Fetal Magnetocardiography (fMCG) has emerged as a highly accurate technique for noninvasive assessment of fetal cardiac electrophysiology. Unlike conventional ECG-based methods, fMCG records the magnetic fields generated by cardiac electrical activity, which are substantially less affected by surrounding biological tissues. This results in higher-quality waveforms and more reliable assessment of fetal cardiac conduction than fetal surface electrocardiography [5,9-12]. Previous studies and systematic reviews have consistently demonstrated the ability of fMCG to detect a broad spectrum of fetal arrhythmias, including QT interval prolongation suggestive of congenital LQTS. In contrast, fetal echocardiography is widely available in routine prenatal care because it is safe, noninvasive, free of ionizing radiation, and routinely performed in most obstetric centers. Therefore, if sufficient diagnostic accuracy can be achieved, echocardiography may represent a practical first-line screening modality in settings where fMCG is unavailable [5,9,13,14].

Although fMCG is widely regarded as the reference standard for fetal electrophysiological assessment, its clinical application remains limited because of its high cost, requirement for magnetically shielded facilities, and availability only in a small number of specialized centers worldwide. Previous investigations evaluating echocardiographic mechanical surrogates of QT and PR intervals have generally been constrained by small sample sizes, limited gestational age ranges, lack of systematic comparison with gestational age-specific fMCG reference values, and insufficient evaluation of diagnostic performance. These limitations have restricted the clinical implementation of echocardiographic screening for fetal LQTS. To address these limitations, the present study was designed to evaluate the feasibility and diagnostic performance of a standardized pulsed-wave Doppler echocardiography protocol in a large prospective cohort of unselected fetuses using gestational age-adjusted fMCG reference values. The primary objective was to determine whether standardized pulsed-wave Doppler echocardiography could provide reliable measurements of fetal QT and PR intervals. In addition, the diagnostic accuracy, sensitivity, specificity, and overall clinical utility of this approach were assessed to determine its potential role in prenatal screening for congenital long QT syndrome and other fetal cardiac conduction abnormalities.

## 2 METHODOLOGY

### 2.1 Study Design and Participants

This cross-sectional analytical study was conducted at the Fetal Echocardiography Unit of Imam Reza Hospital, Mashhad University of Medical Sciences, Iran, between 2022 and 2023. A total of 576 pregnant women with singleton pregnancies between 16 and 38 weeks of gestation were enrolled. Participants underwent routine prenatal screening or detailed fetal cardiac evaluation based on clinical indications. The analysis focused on fetal cardiac measurements, with gestational age considered the primary demographic variable. Sample size estimation was based on an anticipated moderate-to-strong correlation (r = 0.60) between Doppler-derived and Fetal Magnetocardiography (fMCG)-derived cardiac intervals, with a significance level of α = 0.05 and a statistical power of 90%. The calculated minimum sample size was 85 participants. The final sample of 576 participants substantially exceeded this requirement, providing statistical power greater than 95% for agreement analyses and evaluation of diagnostic performance. Fetuses with structural congenital heart disease were excluded from the study.

### 2.2 Data Collection and Echocardiographic Assessment

All examinations were performed using GE Vivid E9 or Philips EPIQ 7 ultrasound systems operating in pulsed-wave Doppler mode. Fetal Heart Rate (HR), PR interval, and QT interval were measured according to a standardized imaging protocol. To minimize measurement variability, all recordings were obtained during fetal quiescence, defined as the absence of fetal breathing movements and body motion.

### 2.3 Measurement Technique

The PR interval was defined as the time from the onset of the mitral valve A-wave, representing atrial contraction, to the onset of ventricular ejection in either the aortic or pulmonary outflow tract. The mechanical surrogate for the QT interval was defined as the interval between the end of the mitral A-wave and the onset of the subsequent mitral E-wave in the following cardiac cycle, as previously validated. This approach is based on atrioventricular valve flow patterns that serve as mechanical correlates of the fetal electrophysiological QT interval.

### 2.4 Waveform Definitions

A-wave: Late diastolic ventricular filling resulting from atrial contraction.

Ventricular ejection wave: Systolic blood flow from the ventricles into the great arteries.

Reference Standard: Fetal Magnetocardiography (fMCG).

Echocardiographically measured PR and QT intervals were compared with published gestational age-adjusted normative reference values obtained from fetal magnetocardiography (fMCG) studies [15-17]. In addition, a subset of 42 fetuses underwent paired fMCG assessment within 48 hours of echocardiography using a 64-channel biomagnetometer system (CTF Systems Inc., Canada) in a magnetically shielded room to enable direct method comparison. Interobserver and intraobserver variability were not formally evaluated in this study.

### 2.5 Statistical Analysis

Statistical analyses were performed using IBM SPSS Statistics version 24.0. Data normality was assessed using the Kolmogorov-Smirnov test. Because fetal heart rate, PR interval, and QT interval were not normally distributed (all *p* < 0.001), nonparametric statistical methods were applied throughout the analysis.

Agreement between pulsed-wave Doppler echocardiography and fMCG was evaluated using Bland-Altman analysis, including calculation of the mean bias and 95% limits of agreement, together with the intraclass correlation coefficient (ICC; two-way mixed-effects model with absolute agreement) in the paired validation subset (n = 42). For the entire cohort (N = 576), Doppler-derived measurements were compared with published gestational age-specific fMCG reference ranges using nonparametric statistical methods, with the Runs test performed as an additional analysis.

Associations between gestational age and Doppler-derived PR and QT intervals were assessed using Spearman's rank correlation coefficient. Diagnostic performance for detecting prolonged PR and QT intervals, defined as values above the 95th percentile of gestational age-adjusted fMCG reference values, was evaluated by calculating sensitivity, specificity, Positive Predictive Value (PPV), and Negative Predictive Value (NPV). All statistical tests were two-sided, and a *p*-value < 0.05 was considered statistically significant.

## 3 RESULTS

A total of 576 pregnant women with singleton pregnancies between 16 and 38 weeks of gestation were included in the study. Standardized pulsed-wave Doppler echocardiography was successfully completed in all participants, and fetal heart rate, PR interval, and QT interval were obtained for every fetus. No examinations were excluded because of inadequate image quality.

### 3.1 Gestational Age Distribution

The majority of fetuses were examined during the late third trimester. The distribution of gestational age is presented in Table **1**.

The mean, standard deviation, and range of the measured parameters obtained by pulsed-wave Doppler echocardiography are presented in Table **2**.

### 3.2 Comparison with Fetal Magnetocardiography (fMCG)

A paired analysis was performed in a subset of 42 fetuses that underwent fMCG within 48 hours of their echocardiogram. There was strong agreement between the two modalities in this validation subset. Bland-Altman analysis showed:

Mean bias for PR interval: +2.1 ms (95% limits of agreement: -8.4 to +12.6 ms)

Mean bias for QT interval: +6.8 ms (95% limits of agreement: -18.5 to +32.1 ms)

The intraclass correlation coefficients (ICC, two-way mixed effects, absolute agreement) were 0.89 (95% CI: 0.81-0.94) for the PR interval and 0.82 (95% CI: 0.72-0.89) for the QT interval, indicating good to excellent reliability.

### 3.3 Population-level Comparison (Full Cohort, N = 576)

Doppler-derived measurements showed good overall agreement with published gestational age-adjusted normative fMCG values. There was no statistically significant difference in PR interval (*p* = 0.301) or fetal heart rate (*p* = 0.064) using the non-parametric Runs test. A small but statistically significant difference was observed for the QT interval (*p* = 0.017); however, the magnitude of this difference (approximately 7 ms) was not clinically meaningful.

### 3.4 Diagnostic Performance

Diagnostic performance parameters (sensitivity, specificity, Positive Predictive Value [PPV], and Negative Predictive Value [NPV]) with 95% confidence intervals were calculated using the Wilson score method [18]. Performance was assessed using the criterion of “prolonged” intervals, defined as values above the 95th percentile of gestational age-adjusted fMCG normative data.

Sensitivity: 85%

Specificity: 78%

Positive Predictive Value (PPV): 68%

Negative Predictive Value (NPV): 90%

These results indicate that pulsed-wave Doppler echocardiography is a useful screening tool, particularly for ruling out prolonged intervals in the general pregnant population, given its high negative predictive value.

### 3.5 Effect of Gestational Age

Fetuses were stratified into three groups according to gestational age. The mean values of PR interval, QT interval, and fetal heart rate for each group are presented in Table **3**.

## 4 DISCUSSION

One of the classic inherited cardiac channelopathies is congenital Long QT Syndrome (LQTS). Mutations in genes encoding cardiac ion channels disrupt the normal action potential, leading to delayed ventricular repolarization and prolongation of the QT interval on the electrocardiogram or magnetocardiogram. The prevalence of congenital LQTS is estimated at approximately 1 in 2000 live births (0.05%) [19]. However, some newborn screening studies have reported a higher prevalence, reaching up to 1 in 2500 in apparently healthy infants. The main clinical concerns are the significantly increased risk of life-threatening ventricular arrhythmias, including torsades de pointes, syncope, overlapping cardiomyopathy phenotypes, and sudden cardiac death from infancy to adulthood [1, 20, 21]. While the postnatal clinical features of LQTS are well described, fetal manifestations have received increasing attention over the past decade. Notably, one large study suggested that LQTS-associated mutations may account for up to 10% of stillbirths in otherwise normal pregnancies [22], underscoring the importance of improved prenatal detection. Technical challenges have historically been the primary obstacle to fetal electrophysiological assessment. Maternal tissues, amniotic fluid, and vernix caseosa create high electrical impedance and significant signal attenuation, rendering conventional fetal ECG unreliable for precise measurement of cardiac time intervals [5, 9, 23]. Fetal Magnetocardiography (fMCG) has substantially advanced the field by directly measuring the magnetic fields generated by cardiac electrical activity. This approach minimizes interference from surrounding tissues and produces high-fidelity waveforms suitable for accurate analysis of PR and QT intervals [17, 24, 25]. The reliability of fMCG for the diagnosis and management of fetal arrhythmias, including LQTS, has been confirmed in multiple studies and systematic reviews [15, 26]. In clinical practice, fMCG has been shown to influence management decisions-such as initiation of transplacental antiarrhythmic therapy, intensified surveillance, or optimization of delivery timing-in 15-75% of fetuses with suspected rhythm disturbances [27, 28]. Nevertheless, fMCG remains limited to a few tertiary centers worldwide due to the need for specialized equipment and magnetically shielded rooms, which are expensive [24, 29]. This restricted availability provided the rationale for the present study. We therefore evaluated pulsed-wave Doppler echocardiography as a practical and widely accessible alternative for measuring fetal PR and QT intervals using validated mechanical surrogates. The objective was to determine whether this commonly used imaging modality provides sufficient agreement with the reference standard (fMCG) to be applied in routine prenatal clinical practice. Fetal heart rate monitoring remains one of the fundamental tools in obstetric medicine for the early detection of perinatal complications. Current non-invasive techniques include Doppler ultrasound, fMCG, and fetal ECG [15, 30]. Among these, fMCG offers excellent accuracy and signal quality for electrophysiological interval measurement [13, 16, 20]. However, its limited availability restricts its use to highly selected cases. In contrast, pulsed-wave Doppler echocardiography is nearly universally available in prenatal clinics and is routinely used to assess fetal heart rate from valvular flow patterns. Although concerns exist regarding cumulative ultrasound exposure and potentially lower temporal resolution compared with direct electrical recording, Doppler echocardiography is considered safe and practical for routine clinical use [31-35].

The findings of the current study strongly support the clinical utility of this approach. There was overall good agreement between Doppler-derived PR and QT intervals and fMCG reference standards. Bland-Altman analysis of the paired validation subset demonstrated minimal systematic bias, with clinically acceptable limits of agreement. Intraclass correlation coefficients indicated good to excellent reliability for both intervals. The technique also showed good sensitivity and specificity for detecting prolonged intervals across the entire cohort. Importantly, there were no clinically meaningful differences in PR interval or fetal heart rate, and measurements remained consistent across a wide range of gestational ages. These results support the potential role of standardized pulsed-wave Doppler echocardiography as a practical screening tool for assessing fetal cardiac repolarization and conduction abnormalities in routine prenatal care. The mechanical surrogate used in this study is the time interval from the end of the mitral A-wave to the onset of the next E-wave, representing a surrogate for the electrical QT interval. Although a small electromechanical delay exists between electrical repolarization and mechanical relaxation, previous validation studies have shown good correlation between this Doppler-derived interval and direct electrophysiological measurements in fetuses. This electromechanical delay remains an inherent limitation of mechanical surrogates; therefore, future studies incorporating simultaneous electrical and mechanical recordings would be valuable.

## 5 STUDY LIMITATIONS

This study has several limitations. First, logistical constraints permitted contemporaneous fMCG recordings in only a subset of 42 fetuses. Consequently, test performance in the remaining fetuses was assessed using published normative reference values. Second, raw QT intervals were analyzed rather than heart rate-corrected QTc intervals, as no universally accepted correction formula exists for fetal QT intervals. Third, postnatal confirmation of diagnoses was unavailable, and long-term arrhythmic outcomes were not assessed. Finally, as a single-center study, multicenter validation is needed to confirm the generalizability of the results across different populations and ultrasound systems.

## CONCLUSION

This prospective study demonstrates that Pulsed-Wave (PW) Doppler echocardiography is a reliable method for measuring fetal PR and QT intervals, with good agreement with fMCG reference standards. The technique offers good diagnostic performance for detecting prolonged intervals and represents a practical, widely accessible, and non-invasive approach for prenatal screening of fetal cardiac repolarization and conduction abnormalities-particularly in settings where fMCG is unavailable. Wider adoption of this method may facilitate earlier identification of congenital LQTS, potentially reducing unexplained stillbirths and sudden infant deaths through improved risk stratification and perinatal management.

## Figures and Tables

**Table 1:** Gestational age distribution of the study population (N = 576).

**GA Group**	**N**	**Percentage**
<32 weeks	107	18.6%
32-37 weeks	21	3.6%
>37 weeks	448	77.8%
**Total**	**576**	**100%**

**Table 2:** Descriptive statistics of fetal heart rate and intervals measured by pulsed-wave doppler echocardiography (N = 576).

**Variable**	**Mean**	**SD**	**Minimum**	**Maximum**	**25th Percentile**	**Median**	**75th Percentile**
Heart Rate (bpm)	146.5	12.8	100.0	174.8	140.0	146.0	152.0
PR Interval (ms)	119.3	16.2	110.0	323.0	110.0	120.0	127.0
QT Interval (ms)	270.0	38.8	133.0	280.0	247.0	263.0	283.0

All variables were found to be non-normal distributed by the Kolmogorov-Smirnov test (*p* < 0.001). Thus, all subsequent analysis was based on non-parametric statistical methods.

**Table 3:** Fetal cardiac parameters stratified by gestational age group.

**GA Group**	**HR (bpm)**	**PR Interval (ms)**	**QT Interval (ms)**
<32 weeks	145.7	118.4	272.9
32-37 weeks	135.8	122.9	289.3
>37 weeks	146.3	120.0	271.3

There was no statistically significant correlation with gestational age in the range studied for PR interval (*p* > 0.05), QT interval (*p* > 0.05), or fetal heart rate (*p* > 0.05) as determined by Spearman correlation analysis.

## Data Availability

The data that support the findings of this study are available within the article.

## LIST OF ABBREVIATIONS

bpm: Beats Per Minute
CI: Confidence Interval
DUS: Doppler Ultrasound
ECG: Electrocardiogram
fHR: Fetal Heart Rate
fMCG: Fetal Magnetocardiography
fsECG: Fetal Scalp Electrocardiography
GA: Gestational Age
HR: Heart Rate
ICC: Intraclass Correlation Coefficient
LQTS: Long QT Syndrome
MCG: Magnetocardiography
ms: Milliseconds
NPV: Negative Predictive Value
PPV: Positive Predictive Value
PR Interval: PR Interval
PWDE: Pulsed-Wave Doppler Echocardiography
PW Doppler: Pulsed-Wave Doppler Echocardiography
QT Interval: QT Interval
QTc: Corrected QT Interval
SPSS: Statistical Package for the Social Sciences
TdP: Torsade de Pointes

## STANDARDS OF REPORTING

STROBE guidelines were followed.
